# Metabolite profiling and in-silico studies show multiple effects of insecticidal actinobacterium on *Spodoptera littoralis*

**DOI:** 10.1038/s41598-024-53096-y

**Published:** 2024-02-06

**Authors:** Mohamed Khaled Diab, Hala Mohamed Mead, Mohamad Ahmad Khedr, Mohamed S. Nafie, Abdelghafar Mohamed Abu-Elsaoud, Sahar Ahmed El-Shatoury

**Affiliations:** 1 Agricultural Research Center, Pest Physiology Department, Plant Protection Research Institute, Giza, 12311 Egypt; 2 Agricultural Research Center, Cotton Leafworm Department, Plant Protection Research Institute, Giza, 12311 Egypt; 3 Faculty of Science, Chemistry Department, Suez Canal University, Ismailia, 41522 Egypt; 4 Faculty of Science, Microbiology & Botany Department, Suez Canal University, Ismailia, 41522 Egypt

**Keywords:** Biochemistry, Chemical biology, Computational biology and bioinformatics, Drug discovery, Microbiology

## Abstract

The polyphagous pest, *Spodoptera littoralis* (Boisduval), poses a significant global economic threat by gregariously feeding on over a hundred plant species, causing substantial agricultural losses. Addressing this challenge requires ongoing research to identify environmentally safe control agents. This study aimed to elucidate the insecticidal activity of the metabolite (ES2) from a promising endophytic actinobacterium strain, *Streptomyces* sp. ES2 EMCC2291. We assessed the activity of ES2 against the eggs and fourth-instar larvae of S. littoralis through spectrophotometric measurements of total soluble protein, α- and β-esterases, polyphenol oxidase (PPO), and catalase enzyme (CAT). The assessments were compared to commercial Biosad® 22.8% SC. Untargeted metabolomics using LC-QTOF-MS/MS identified 83 metabolic compounds as chemical constituents of ES2. The median lethal concentration (LC50) of ES2 (165 mg/mL) for treated *Spodoptera littoralis* eggs showed significant differences in polyphenol oxidase and catalase enzymatic activities, while the LC50 of ES2 (695 mg/mL) for treated *S. littoralis* fourth instar larvae showed lower significance in α- and β-esterase activities. Molecular docking of ES2 identified seven potent biocidal compounds, showing strong affinity to PPO and catalase CAT proteins in *S. littoralis* eggs while displaying limited binding to alpha and beta esterase proteins in the larvae. The results contribute to the understanding of ES2 as a promising alternative biopesticide, providing insights for future research and innovative applications in sustainable pest management strategies.

## Introduction

Microbial secondary metabolites emerge as a promising sustainable source for new opportunities of application, emphasizing their potential for several agricultural purposes^1^. Actinobacteria, in particular, effectively enhance the overall durability of plants in agricultural and ecological settings by synthesizing secondary metabolites and enzymes that serve as natural defenses against damaging pests and diseases^2^. Natural insecticides were derived from soil actinobacteria by fermentation procedures, including avermectins, spinosyns, and milbemycins^3–5^. *Streptomyces* is the largest producer genus of known secondary metabolites of actinomycetes that produce 70–80% of bioactive natural products^6^. *Streptomyces mutabilis* IMA8 produces a chitinase enzyme that has a larvicidal effect against the larval and pupal stages of *Anopheles stephensi* and *Aedes aegypti* (Diptera)^7^.

The herbivorous cotton leafworm, *Spodoptera littoralis* (Boisduval), attacks more than a hundred plant species annually by feeding gregariously on its shoot system, causing massive economic losses^8^. It is considered a global economic threat^9^. The management of *S. littoralis* to ensure stable and high crop output is a great challenge that necessitates permanent and continuous research to find renewable and innovative control agents^10^. Efforts to limit insect spread and reduce damage should consider environmental considerations^11,12^.

Untargeted metabolomics is an analytical approach utilized to identify and analyze chemical constituents of metabolites without prior knowledge of their specific identity^13^. Recently, it has been increasingly used to identify microbial metabolites, which are promising sources of bioactive compounds with potential applications in medicine, agriculture, and industry^14–17^. Its application in fungal research has helped in five aspects: identification, response to stress, metabolite discovery, metabolism engineering, and fungal interactions with plants^18^. Untargeted metabolomics and bioinformatics were applied to set up biomarkers of aflatoxigenic *Aspergillus* species^19^. In drug discovery field, the approach of combining metabolomics with molecular docking is successfully applied to investigate the mechanisms of action of natural compounds derived from microorganisms ^20^. Molecular docking has determined the inhibition types and binding modes of α-glucosidase from two mangrove-derived actinomycetes, *Streptomyces* sp. WHUA03267 and *St.* sp. WHUA03072^21^. Additionally, molecular docking was used to assess insecticidal activity against *Plutella xylostella* and illustrate the identified compound action with the GABA (γ-aminobutyric acid) receptor^21^.

Measuring the enzymatic activities in insects can provide valuable information on the effectiveness of insecticide treatments and the potential for the development of resistance^22,23^. Nonspecific esterases (NSEs) are an enzymatic class that play an important role in xenobiotic metabolism, including insecticides^24^. In insects, NSEs are classified into two groups: alpha and beta, responsible for insecticide detoxification and insecticide resistance development. The detoxification enzyme activity (α and β esterase) was measured for *Aedes aegypti* and *Spodoptera litura* exposed to different concentrations of biosynthesized TiO_2_ nanoparticles^25^. Polyphenol oxidase (PPO) and catalase (CAT) are two enzymes that play important roles in defense mechanisms. PPO is involved in toxic quinone production from phenolic compounds, while CAT is involved in hydrogen peroxide breakdown^26^. Abdel-Aziz’s research team have proved the role of PPO and CAT in the defense response on *Citrus aurantium* against *Parlatoria ziziphi*^27^.

This research intended to shed light on the activity of the endophytic actinobacterium *Streptomyces* ES2 EMCC2291 as a pesticide agent and to demonstrate its chemical profile. Moreover, this study contributes to the systematic evaluation and application of crude ES2 extract as an ovicidal and larvicidal agent against the cotton leafworm *S. littoralis*. Our study illustrates endophytes as a prospective future source that can be relied upon in the field of pest control.

## Results

### Metabolite profiling of ES2 crude extract using LC-QTOF-MS/MS

**Figure 1:**
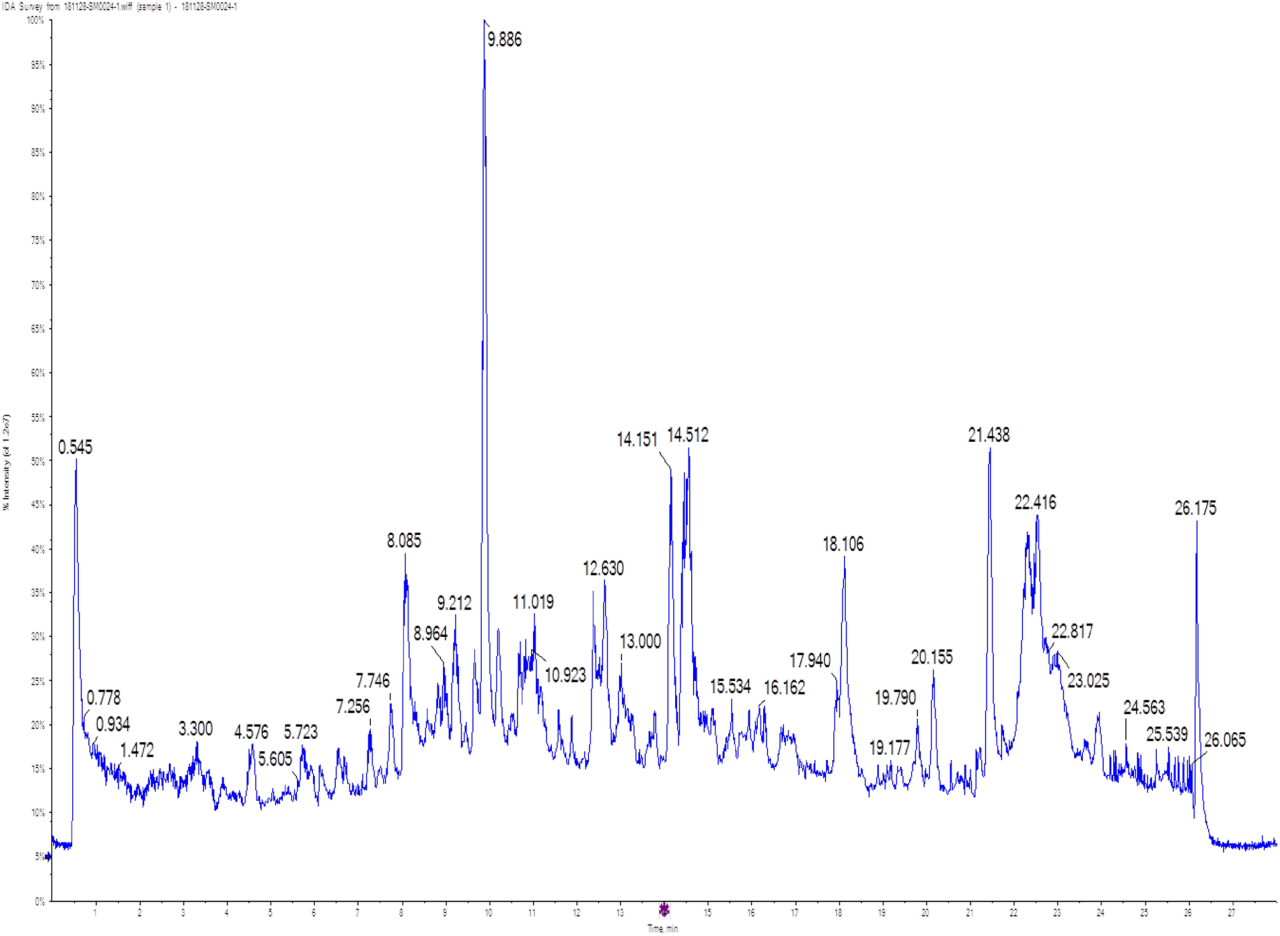
Positive mode total identified chromatogram for *Streptomyces* ES2 EMCC2291 ethyl acetate metabolic extract using LC-QTOF-MS/MS. The total identified chromatogram (TIC) represents the main raw dataset compared to the built-in database (Data Acquisition Analyst TF 1.7.1 software, Sciex) and online databases. The chromatogram is shown as the peak relative retention time. LC-QTOF-MS/MS, liquid chromatography, combined with quadrupole-time-of-flight high-definition mass spectrometry instrument.

Untargeted metabolomics is a method that aims to comprehensively analyze all metabolites present in a sample, without prior knowledge of their chemical identity. The untargeted metabolomics analysis, using the LC-QTOF-MS/MS technique, identified 83 metabolic compounds (Supplementary Table S1 online) from the complete metabolite profiling of ES2 crude extract (Fig. 1). The identified compounds belonged to 15 chemical classes: phenol (17), acid (13), aromatic amine (12), amide (7), ester (7), alcohol (5), aliphatic amine (5), α, β unsaturated carbonyl compound (5), amino acid (3), ketone (3), aliphatic hydrocarbon (2), aliphatic ether (1), sulfur-containing compound (1), anhydride (1), and phosphorous compound (1). Seven compounds were identified as key constituents of the ES2 metabolic extract. These were 4-nitrophenol, Cyromazine, Di (2-ethylhexyl) phthalate (DEHP), Jasmonic acid, Diazinon, Dioctyl Phthalate, and Brevicompanine B (Table 1). The acquired data from LC-QTOF-MS/MS libraries were compared to the five most common chemical databases: ChemSpider ID, Chemical structure database identifier (http://www.chemspider.com/); InChI key, International chemical identifier (https://pubchem.ncbi.nlm.nih.gov/source/ChEBI); KEGG ID, Kyoto encyclopedia of genes and genomes (https://www.genome.jp/kegg/); METLIN ID, METLIN metabolite and chemical entity database (https://metlin.scripps.edu/); and CAS registry number, chemical abstracts service database (https://www.cas.org/). The data were identified with a ratio of more than 99.9% compared to the built-in database (Data Acquisition Analyst TF 1.7.1 software, Sciex) and online databases (MassBank of North America “MoNA”, Yeast Metabolome database “YMDB”, EcoCyc *E. coli* database, Human Metabolome database “HMDB”, and DrugBank database).

**Table 1:** Key constituents of *Streptomyces* ES2 EMCC2291 ethyl acetate metabolic extract.

No	RT (min.)	Intensity	Mass (Da)	Adduct	m/z value (mass)*	Molecular formula	Identified compound	InChI Key	Chemical structure	Chemical class	ChemSpider ID*, KEGG ID**, PubChem CID***, METLIN ID****, CAS Registry Number#
1	0.9714	12,436.39	139.1088	[M + H]^+^	140.1089	C_6_H_5_NO_3_	4-Nitrophenol	BTJIUGUIPKRLHP-UHFFFAOYSA-N		Phenol	955*, C00870**, 980***, 4100****, 100–02-7#
2	3.4676	56,928.55	166.09669	[M + H]^+^	167.1178	C_6_H_10_N_6_	Cyromazine	LVQDKIWDGQRHTE-UHFFFAOYSA-N		Aromatic amine	43,550*, C14147**, 47,866***, NA****, 66,215–27-8#
3	6.3135	5132.789	390.27701	[M + H]^+^	391.1884	C_24_H_38_O_4_	Di(2-ethylhexyl) phthalate (DEHP)	BJQHLKABXJIVAM-UHFFFAOYSA-N		Ester	21,106,505*, C03690**, 8343***, NA****, 117–81-7#
4	6.8179	14,005.92	210.1256	[M + H]^+^	211.1690	C_12_H_18_O_3_	Jasmonic Acid	ZNJFBWYDHIGLCU-UHFFFAOYSA-N		Acid	4,444,606*, C08491**, 5,281,166***, NA****, 221,682–41-3#
5	11.0200	6871	166.09669	[M + H]^+^	167.10397	C_6_H_10_N_6_	Cyromazine	LVQDKIWDGQRHTE-UHFFFAOYSA-N		Aromatic amine	43,550*, C14147**, 47,866***, NA****, 66,215–27-8#
6	13.0200	1941	304.10105	[M + H]^+^	305.10833	C_12_H_21_N_2_O_3_PS	Diazinon	FHIVAFMUCKRCQO-UHFFFAOYSA-N		Aromatic amine	2909*, C14324**, 3017***, NA****, 333–41-5#
7	21.4234	1,228,978	390.27701	[M + H]^+^	391.283	C_24_H_38_O_4_	Dioctyl Phthalate	MQIUGAXCHLFZKX-UHFFFAOYSA-N		Ester	8043*, C03690**, 8346***, NA****, 68,515–43-5#
8	23.1000	6099	367.22598	[M + H]^+^	368.22598	C_22_H_29_N_3_O_2_	Brevicompanine B	HAXPBJUEOMQIJN-UHFFFAOYSA-N		Di-ketone	8,960,513*, NA**, 51,340,320***, NA****, 215,121–47-4#

* refers to ChemSpider ID; ** refers to KEGG ID; *** refers to PubChem CID; **** refers to METLIN ID; # refers to CAS Registry Number.

Mass (Da); molecular weight in Dalton. (m/z) are values detected by mass spectrometry; RT, retention time; NA, not available.

Chemical structures were carried out using Reaxys ChemDraw software, version 18.0.0.20 (https://www.reaxys.com.mplbci.ekb.eg/chemdrawservices/restn).

LC-QTOF-MS/MS, liquid chromatography, combined with quadrupole-time-of-flight high-definition mass spectrometry instrument.

CAS Registry Number, Chemical Abstracts Service database (https://www.cas.org/); ChemSpider ID, Chemical structure Database Identifier (http://www.chemspider.com/); Errors (ppm) were obtained by formula prediction software in the mass spectrometer; InChI Key, International Chemical Identifier (https://pubchem.ncbi.nlm.nih.gov/source/ChEBI); KEGG ID, Kyoto Encyclopedia of Genes and Genomes (http://www.genome.jp/kegg); METLIN ID, METLIN Metabolite and Chemical Entity Database (https://metlin.scripps.edu/); PubChem CID, A Database of Chemical Molecules and Their Activities against Biological Assays (https://pubchem.ncbi.nlm.nih.gov/).

### Biochemical assessments for nonspecific esterases of treated S. littoralis fourth instar larvae

Alpha esterase enzymatic activity at normal (distilled water), negative control (ethyl acetate), Biosad® 22.8%, and ES2 Metabolite showed an average (± SE) of 13.266 ± 0.425, 13.966 ± 0.463, 10.033 ± 0.088, and 10.433 ± 0.233 µg α-naphthol/min/mg protein. Alpha esterase activity in Biosad® 22.8% (10.033 ± 0.088) was significantly decreased compared with the normal control (13.266 ± 0.425) and negative control (13.966 ± 0.463), as revealed by DMRTs at the 0.05 level. In addition, alpha esterase activity in the ES2 metabolite (10.433 ± 0.233) was significantly decreased versus the normal control (13.266 ± 0.425) and negative control (13.966 ± 0.463), as revealed by DMRTs at the 0.05 level (Table 2). Beta esterase activities in Biosad® 22.8% showed an average of 9.233 ± 0.12 µg β-naphthol/min/mg protein, which is significantly lower than that of the normal control (11.1 ± 0.208) and negative control (12.233 ± 0.202), as revealed by DMRTs. Furthermore, beta esterase activities in the ES2 metabolite group (11.133 ± 0.176) showed no significant difference versus the normal control; however, a significant difference against the negative control was observed, as revealed by ANOVA and DMRTs.

**Table 2:** Alpha and beta esterase activities of *Spodoptera littoralis* fourth instar larvae treated with *Streptomyces* ES2 metabolites.

Treatment	α esterase (µg α- naphthol/min/mg protein)	β esterase (µg β- naphthol/min/mg protein)
Normal distilled water	13.266 ± 0.425a	11.1 ± 0.208b
Negative Control Ethyl acetate	13.966 ± 0.463a	12.233 ± 0.202a
Biosad® 22.8% SC (LC_50_: 0.17 mL/L)	10.033 ± 0.088b	9.233 ± 0.12c
*Streptomyces* ES2 Metabolite (LC_50_: 695 mg/mL)	10.433 ± 0.233b	11.133 ± 0.176b
ANOVA- 1 way		
F-ratio	52.643	69.452
*p*-value	< 0.001 ***	< 0.001***

Data are expressed as the mean ± S.E.; the mean under each variety with different letters in the same column denotes a significant difference according to DMRTs at *p* ≤ 0.05. *** = *p* ≤ 0.01.

Normal, Control was treated with distilled water for experimental adjustment; Negative Control, control was treated with ethyl acetate solvent for experimental adjustment; *Streptomyces* ES2 Metabolite, an actinobacterial crude metabolite produced by *Streptomyces* ES2 EMCC2291; F-ratio, Frequency ratio.

All biochemical measurements were performed in triplicate.

Biosad® 22.8% SC was used at an LC_50_ rate of 0.17 mL/L.

The *Streptomyces* ES2 crude metabolite was used at its LC_50_ at a concentration of 695 mg/mL.

### Biochemical assessments for polyphenol oxidase and catalase activities of treated S. littoralis egg masses

Biochemical assessments of laboratory *S. littoralis* egg masses treated with crude ES2 metabolites; in particular, polyphenol oxidase and catalase enzyme activities present ovicidal biological effects. Polyphenol oxidase enzyme activities in Biosad® 22.8% showed an average of 1748 ± 13.316 m ∆ O.D./min/mg protein, which is significantly higher than the normal control (414.333 ± 12.732) by 321.9% and the negative control (428.666 ± 16.414 m ∆ O.D./min/mg) by 307.8%, as revealed by DMRTs. Furthermore, treatment with the ES2 metabolite group significantly increased PPO activity by Biosad® 22.8% (509.0 ± 8.50), as revealed by ANOVA and DMRTs. On the other hand, Biosad® 22.8% significantly lowered CAT activities by 10.2% and ES by 5.3% from the normal control as revealed by DMRTs at 0.05 (Table 3).

**Table 3:** Polyphenol oxidase and catalase enzyme activities of *Spodoptera littoralis* egg masses treated with *Streptomyces* ES2 metabolites.

Treatment	Polyphenol oxidase (PPO) (m ∆ O.D./min/mg protein)	Catalase (CAT) (mU/mg protein)
Normal distilled water	414.333 ± 12.732c	228 ± 4.725a
Negative Control Ethyl acetate	428.666 ± 16.414c	222.333 ± 3.844ab
Biosad® 22.8% SC (LC_50_: 0.033 mL/L)	1748 ± 13.316a	204.666 ± 3.711c
*Streptomyces* ES2 Metabolite (LC_50_: 165 mg/mL)	509 ± 8.504b	216 ± 2.516b
ANOVA- 1 way		
F-ratio	992.86	6.841
*p*-value	< 0.0001 ***	0.0064 **

Data are expressed as the mean ± S.E.; the mean under each variety with different letters in the same column denotes a significant difference according to DMRTs at *p* ≤ 0.05. *** = *p* ≤ 0.01.

Normal, Control was treated with distilled water for experimental adjustment; Negative Control, control was treated with ethyl acetate solvent for experimental adjustment; *Streptomyces* ES2 Metabolite, an actinobacterial crude metabolite produced by *Streptomyces* ES2 EMCC2291; F-ratio, Frequency ratio.

All biochemical measurements were performed in triplicate.

Biosad® 22.8% SC was used at an LC_50_ rate of 0.033 mL/L.

The *Streptomyces* ES2 crude metabolite was used at its LC_50_ at 165 mg/mL.

### Molecular docking simulation

**Figure 2:**
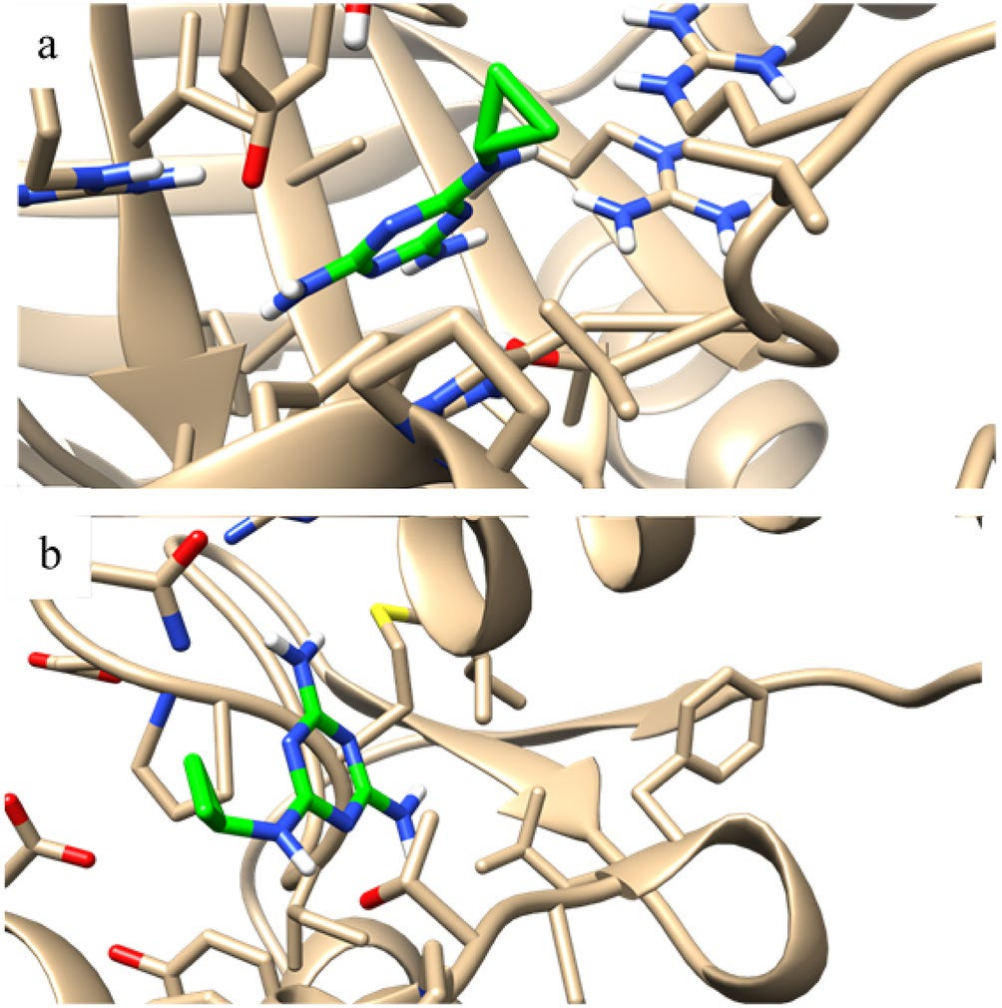
Binding disposition and ligand-receptor interactions of cyromazine toward polyphenol oxidase and catalase active sites. Cyromazine is shown in green; a, polyphenol oxidase (PPO) and b, catalase (CAT) active sites are shown in black. Hereto-atoms of oxygen, nitrogen, and hydrogen with standard colors. Molecular docking studies were carried out using AutoDock Vina version 4 (https://vina.scripps.edu/) as the computational software.

Molecular docking was conducted for the seven major compounds in ES2 metabolites to illustrate the virtual binding mechanism inside polyphenol oxidase and catalase proteins. The results explained the mode of action inside the treated *S. littoralis* egg cells. As summarized in Table 4, all the compounds exhibited good binding affinity toward the tested proteins with good binding energy (negative values -26.18 to -2.1 kcal/mol), except DEHP and brevicompanine B compounds for CAT protein with positive values. The compounds exhibited good interaction profiles with the key amino acids of both proteins. The three-dimensional visualization in Fig. 2 shows that cyromazine (with the lowest binding energy) was deposited inside the binding sites of polyphenol oxidase and catalase proteins and formed good interactive profiles with the key amino acids.

**Table 4:** Molecular docking for the seven key *Streptomyces* ES2 constituents inside polyphenol oxidase and catalase proteins of *Spodoptera littoralis* eggs.

	Polyphenol oxidase (PPO) (PDB = 3HHS)		Catalase (CAT) (PDB = 2A9E)	
	Binding energy (Kcal/mol)	Ligand‒receptor interactions	Binding energy (Kcal/mol)	Ligand‒receptor interactions
4-nitrophenol	−11.28	1 H-bond with **Glu 166**	−9.56	1 H-bond with **Tyr 339** Arene-cation with **Arg 335** Arene-arene with **Phe 142**
Cyromazine	−10.52	3 H-bonds with **Met 19** and **Thr 38**	−14.68	Arene-arene with **His 56**
Di (2-ethylhexyl) phthalate (DEHP)	−26.18	1 H-bond with **Asn 81**	–	1 H-bond with **Arg 335** Arene-arene with **His 56**
Jasmonic Acid	−16.05	2 H-bonds with **Arg 122**	−5.65	3 H-bonds with **Arg 53**, **Ala 313,** and **Phe 315**
Diazinon	−20.83	1 H-bond with **Asn 81**	−2.1	Arene-arene with **His 56**
Dioctyl Phthalate	−20.45	1 H-bond with **Asn 81**	−3.2	1 H-bond with **Arg 53** Arene-cation with **Arg 93**
Brevicompanine B	−11.11	Arene-cation with **Arg 163**	–	1 H-bond with **Arg 346** Arene-arene with **His 56**

Bold amino acids are the key interactive ones with which native ligands interact. The binding energy of the ligand–protein complex in Kcal/mol.

**2A9E,** Cocrystalized ligand for catalase protein; **3HHS,** Cocrystalized ligand for polyphenol oxidase protein; **PDB,** Protein data bank code; **Ala,** alanine amino acid; **Arg,** Arginine amino acid; **Asn,** Asparagine amino acid; **Glu,** Glutamate amino acid; **His,** Histidine amino acid; **Met,** Methionine amino acid; **Phe,** Phenylalanine amino acid; **Thr,** Threonine amino acid; **Tyr,** Tyrosine amino acid.

Molecular docking studies were carried out using AutoDock Vina version 4 (https://vina.scripps.edu/) as the computational software; PDB, Protein Data Bank (http://www.rcsb.org/) was used to select the targeted proteins’ cocrystalized ligands.

## Discussion

This study was conducted to assess the significance of endophytic actinobacterial secondary metabolites in controlling the devastating pest *S. littoralis*, demonstrating their ovicidal and larvicidal activities and providing valuable insights into their mode of action and potential applications.

The results revealed that the ES2 crude metabolite contains seven unique constituents (brevianamid F, 1-naphthylamine, S-adenosylmethioninaminium, palitanin, phthalic anhydride, galaxolidone, and brevicompanine B) that have not previously been recorded from actinomycetes based on our search using the Derwent innovation database^40^. The crude constituents of ES2 combine a variety of biological activities, including biocontrol, protein inhibitor, neurotoxin, antioxidant, immunostimulant, and anticancer activities. The compound 1-naphthylamine has antitumor^41^, antimicrobial^42^, and biocontrol activities against a lepidopteran pest, *Tryporyza intacta*^43^. S-adenosylmethioninaminium has anticancer activity^44^, whereas Brevianamid F and Palitantin have antimicrobial activity^45,46^. These findings highlight the richness of the studied *Streptomyces* ES2 EMCC2291 strain with a highly bioactive metabolome outcome.

The ES2 metabolite showed an ovicidal action stronger than its larvicidal impact against *S. littoralis*. This may be due to the mode of action of ES2 chemical constituents or because the larval stage is stronger than the egg stage^9^. The LC_50_ values were compatible with the toxicity results, where the LC_50_ of the ES2 metabolite was 165 and 695 mg/mL for ovicidal and larvicidal effects, respectively, in parallel with Biosad® 22.8% LC_50_. The biochemical assessments of ES2 showed a significant decrease in α- and β-esterase activities in the treated *S. littoralis* larvae. These results are confirmed by the molecular docking outcomes. These findings varied as a result of the evaluation of compounds on target proteins, confirming El-Kareem’s study^47^. In contrast, the molecular docking results showed good binding energy (negative values, Kcal/mol) for the antioxidant PPO and CAT target proteins and supported its response to ES2 activity in *S. littoralis* eggs. PPO showed a significantly different increase from controls and a decrease from Biosad® 22.8%, while CAT enzymatic activity showed a slightly significant response. Our results indicated that the potency of ES2 against *S. littoralis* eggs agrees with Wang’s research outcome^26^.

Our outcomes are in agreement with a study that investigated the nematocidal activity of *Streptomyces enissocaesilis* OM182843 against root-knot nematodes (*Meloidogyne incognita*)^48^. Our results thus indicated the potential of ES2 to control different *S. littoralis* stages. On the other hand, Zayed et al. assessed the commercial microbial formulation “effective microorganisms” against *S. littoralis* fourth instar larvae, and they reported its antifeedant activity^49^. ES2, being a crude microbial metabolite with both active and inactive constituents, resulted in LC50 levels of 165 mg/mL for ovicidal effects and 695 mg/mL for larvicidal effects. These higher LC50 values are attributed to the diverse composition of ES2. Previously reported LC50 values reflect the overall impact of the heterogeneous metabolite. Examples include, LC_50_ 467.44 mg/mL of fungal metabolites from *Trichoderma longibrachiatum* towards the third instar larvae of *Aedes aegypti*, and LC_90_ 719.07 mg/mL of *T. virde* towards *Ae. Albopictus*^50^. Our future investigations will explore deeper into understanding the specific contributions of active and inactive constituents within ES2, providing valuable insights for refining its application and optimizing its efficacy in pest control strategies."

In conclusion, our current study provides insights into endophytic actinobacterial secondary metabolites as ovicidal and larvicidal activities against a destructive polyphagous pest, *S. littoralis*. Untargeted metabolomics increases the coverage of the metabolome and introduces a widespread recognition of a multiplatform approach to metabolomics. The untargeted metabolomics and molecular docking studies revealed the mode of action inside the treated *S. littoralis* eggs and larvae. The data are beneficial to researchers interested in natural product discovery, particularly microbiologists who focus on secondary metabolite biosynthesis from microbial origin and its applications, and the direction of research for the development of industrial microbiology. Furthermore, these data could be shared with entomologists interested in pest control and the implementation of biocontrol protocols to overcome insecticide resistance phenomena. The full chemical profiling of ES2 active constituents and their expected targets is in progress using biological, bioinformatics, and molecular docking studies.

## Materials and methods

All chemicals and solvents were purchased from Sigma Aldrich. A commercial pesticide, Biosad® 22.8% SC (Mobedco Co., Jordan), was used in all bioassay investigations as a positive control using its recommended rate (0.1 mL/L). Biosad® 22.8% suspension concentrate, w/v active ingredient Spinosad (A + D), is obtained organically from *Saccharopolyspora spinosa* fermentation, a naturally occurring soil actinobacterium. All experiments were performed with relevant institutional, national and international guidelines and legislation.

### Ethical approval

This work does not contain any studies with human participants but includes studies for insects and all protocols and procedures employed in insect studies were ethically reviewed and approved by the Agriculture Research Center, Plant Protection Research Institute and Suez Canal University's ethical committees. All experiments on tested insects will be conducted according to the guidelines of the Food and Agriculture Organization of the United Nations (FAO).

### Endophytic actinobacterium source

The studied actinobacterium strain was previously recovered from the internal tissues of a wild medicinal plant species, *Artemisia judaica* L. (*F. Asteraceae*), from the World Heritage Site of Saint Catherine (WHS No. 954), South Sinai, Egypt (33°57΄29.8˝E 28°33΄51.9˝N 1545.34 m)^28^, and we have previously proven its larvicidal potency^29^. The strain was provided by the Actinobacteria Laboratory, Suez Canal University, Egypt. The strain was provided as spore suspensions in 20% v/v glycerol (ADWIC, Egypt) at -15 °C and was refreshed on starch casein agar (Sigma‒Aldrich, Germany).

### Actinobacterium fermentation and crude secondary metabolite extraction

The studied strain was grown from 2 µL (4–8 × 10^7^ cfu/mL) of spore suspension in 250 mL shake flasks containing 50 mL starch casein broth media and incubated at 28 ± 2 °C for 21 days with continuous shaking at 100 rpm. After complete growth, the broth cultures were centrifuged at 15,000 rpm and filtered to obtain the active crude metabolites. The filtrates were extracted by the liquid–liquid extraction method three times, with absolute ethyl acetate HPLC grade (Sigma‒Aldrich, Germany) equal volumes for 30 min each time. After extraction, the solvent layers were merged and evaporated to dryness using a rotary evaporator (HS-2005S-N, HAHN SHIN Scientific Co., Korea) at 40 °C. The extract was weighed to obtain one gram of the crude metabolite and preserved at 4 °C for the untargeted metabolomics analysis. For the biological assays, the metabolic extract was redissolved in ethyl acetate (Sigma‒Aldrich, Germany) to prepare 200 mg/mL stock concentrations as outlined^30^.

### Cotton leafworm rearing technique

The laboratory strain of *S. littoralis* used in this trial was established from egg masses collected from an open field in Egyptian habitats and reared for more than 25 generations without being exposed to any insecticide pollution. The strain was maintained under stable circumstances; 26 ± 1 °C and 70 ± 5% R. H. with a light: dark 16:8 h photoperiod. During the larval stage, fresh castor oil plant leaves, *Ricinus communis* L., were collected daily from the plant protection research institute’s garden in Sharqia Governorate, Egypt, with relevant permission; and introduced daily to feed the larvae. With the end of the larval stage approaching and the beginning of the prepupal period, tissue paper or wet sawdust was placed at the rearing jar base to provide a pupation cradle. Fully developed pupae were gathered and preserved in clean jars until the emergence of the adults. A 10% sugar solution was given to newly emerged moths, sexed, and allowed to lay their eggs on paper stripes. The collected eggs were preserved until hatching in other cleaned jars. From this culture, egg masses and fourth instar larvae were submitted for the bioassay tests^31^.

### In vivo* biological assay*

To estimate LC_50_ values of ES2 metabolite on both *S. littoralis* eggs and larvae, newly molted *S. littoralis* fourth instar larvae were starved for 4 – 5 h. Fresh castor leaves were collected, cleaned, and cut into equal and identical disks with the help of a cork borer and then impregnated with the corresponding metabolite concentration using a leaf dipping technique assay, as reported^31^. The toxicity of ES2 metabolites on fourth instar larvae was investigated under laboratory conditions at 25, 50, 100, and 200 mg/mL. Effects of ES2 on the egg stage were investigated under laboratory conditions, at the same above concentrations, using a spraying technique to evaluate their ovicidal activity^11^. Biosad® 22.8% SC was investigated using concentrations of (0.05, 0.1, and 0.2 mL/L).

### Metabolite profiling using LC-QTOF-MS/MS.

LC-QTOF-MS/MS was used to detect the chemical components of crude ES2 metabolites^32^. The LC-QTOF-MS/MS analysis was performed using a Triple TOF® 5600 + , Sciex (ISO9001), Canada; fused two LC columns, an in-line filter disk precolumn (0.5 µm × 3.0 mm; Phenomenex Co., U.S.A.); and an XBridge C18 column (3.5 µm, 2.1 × 50 mm; Waters Co., U.S.A.) maintained at 40 °C. The sample was injected in the positive TOF–MS mode. Mass spectrometry was performed on a Triple TOF® 5600 + system. MasterView was used for feature (peaks) extraction from the total ion chromatogram (TIC) using PeakView 2.2 Software Sciex based on the following criteria: features should have a signal-to-noise greater than five (untargeted analysis). Untargeted peak finding and clustering were analyzed by MarkerView 1.3 software (Sciex). MarkerView was used for feature annotation and removing isotopic peaks. MasterView was used again to identify peaks based on their fragments using a built-in database (Data Acquisition Analyst TF 1.7.1 software, Sciex) and online databases. The structures of the resulting compounds were drawn using the Reaxys ChemDraw program (version:18.0.0.20) that was accessed through the link (https://08129xwbz-1103-y-https-www-reaxys-com.mplbci.ekb.eg/chemdrawservices/rest).

### Biochemical assessments

All biochemical measurements were repeated three times as replicates. A double-beam ultraviolet/visible spectrophotometer (Spectronic 1201, Milton Roy Co., U.S.A.) was used to measure the absorbance of colored substances or metabolic compounds. All enzymatic activities were expressed in enzyme units (EU) per mg protein content.

### Sample preparations for biochemical assessments

Healthy individuals of the fourth instar larvae laboratory strain of *S. littoralis*, which were treated with (LC_50_, 695 mg/mL) ES2 crude metabolite, in addition to controls (H_2_O and ethyl acetate solvent) and Biosad® 22.8% SC (LC_50_, 0.17 mL/L), were picked up after 72 h posttreatment. The collected larvae were transferred to cleaned screw-capped tubes and frozen at −20 °C until examination. Each treated larval group (consisting of five larvae weighing 100—350 mg) was homogenized in distilled water (50 mg/1 mL) using a chilled glass Teflon tissue homogenizer (ST–2 Mechanic-Preczyina, Poland) surrounded with a crushed ice jacket for three minutes. Samples of *S. littoralis* egg masses, which were treated with (LC_50_, 165 mg/mL) of ES2 crude metabolite, in addition to controls (H_2_O and ethyl acetate solvent) and Biosad® 22.8% SC (LC_50_, 0.033 mL/L), were collected in clean tubes 48 h posttreatment to avoid egg hatching and kept frozen at −20 °C until studied in addition to the controls. The frozen egg mass groups (consisting of nearly 1000 eggs weighing 5–7 mg) were homogenized in phosphate-buffered saline (Sigma‒Aldrich, Germany) to extract the total soluble proteins. The homogenate samples for egg masses and larvae were centrifuged at 8000 rpm for 15 min at 5 °C in a refrigerated microcentrifuge. After the deposits were cleared away, the supernatants—also known as enzyme extracts—were put into cleaned screw-capped tubes and frozen at −20 °C until they were needed for biochemical tests. based on Ismail’s method with modifications^33^.

### Total soluble protein assessment

The total protein concentration was determined according to Bradford’s method^34^. Bovine serum albumin (Stanbio Laboratory, Texas, U.S.A.) was used to convert to mg/mL as the standard. The absorbance of the samples was measured at 595 nm using a microplate reader.

### Nonspecific esterase determination

Alpha esterases (α–esterases, EC 3.1.1.1) and beta esterases (β–esterases, EC 3.1.1.2) were determined for the treated *S. littoralis* fourth instar larvae^35^ using α–naphthyl acetate or β–naphthyl acetate as substrates. The samples were measured at 600 and 555 nm absorbance for α- and β–naphthol, respectively. The activity was expressed as[µg naphthol/min/mg protein].

### Polyphenol oxidase (PPO, EC 1.10.3.1) determination

Polyphenol oxidase (PPO) activity was measured for the treated *S. littoralis* eggs using a colorimetric assay as declared by Ishaaya^36^, with minor modifications, based on the oxidation of catechol solution (2%) as a substrate. The absorbance of the samples was measured at 405 nm against a blank. The activity was expressed as [m ∆ O.D./min/mg protein].

### Catalase enzyme (CAT, EC 1.11.1.6) determination

Catalase enzyme (CAT) activity was measured for the treated *S. littoralis* eggs using a spectrophotometric assay, using Biodiagnostic Kit No. SD 2517, which is based on the decomposition of hydrogen peroxide (H_2_O_2_) by CAT, resulting in a decrease in absorbance^37^. The absorbance of the samples was measured at 510 nm against a blank. The activity was expressed as [mU/mg protein].

### Molecular docking simulation

Molecular docking simulation is a computational technique aimed at predicting the binding mode and affinity of a small molecule (ligand) to a target protein. It involves the calculation of the energetics and geometry of the interaction between the ligand and the protein. The technique involves the use of computer algorithms to predict and identify the most energetically favorable binding mode of a small molecule to a target protein, which can then be further optimized and tested in vitro and in vivo. Molecular docking illustrates the virtual mechanism of binding of selected compounds polyphenol oxidase (PPO, PDB = 3HHS), and catalase (CAT, PDB = 2A9E) target proteins. The data were freely accessible through the protein data bank. Both proteins and ligands were optimized, and the molecular docking study was carried out using AutoDock Vina as the computational software^38^. Each complex was analyzed for 3D interaction images taken by Chimera (UCSF)^39^.

### Statistical data analysis

**Figure 3:**
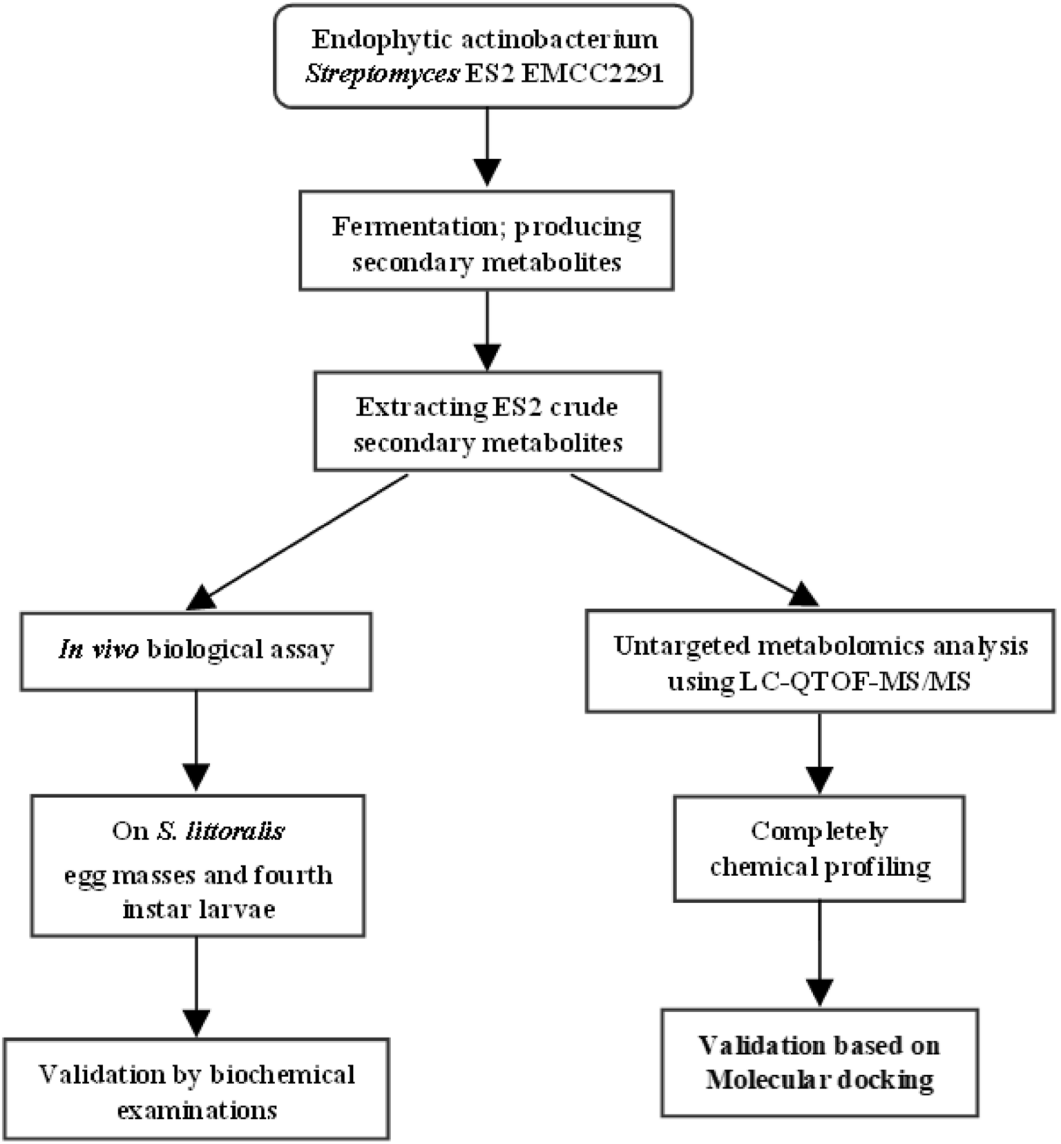
Schematic flowchart illustrating the research steps of the current study.

All extractions and determinations were conducted in triplicate, and the results were expressed based on dry weight (DW). Data are expressed as the mean value (n = 3) ± standard error of the mean (SE). The LC_50_ values were calculated by linear regression analysis. Pearson’s correlation coefficients were computed between secondary metabolites and biocide activity. Normality testing was performed, using Shapiro–Wilk and Kolmogorov–Smirnov at 0.05 level, for detecting parametric and nonparametric variables. The parametric variables included: total protein, α– and β–esterase, and catalase. Phenol oxidases data was nonparametric, and it was represented in a parametric form for better presentation of mean and standard deviation (Supplementary Table S2)*.* Differences between groups were performed using one-way ANOVA and ANOVA followed by Duncan’s Multiple Range Test (DMRTs) to further check the differences between groups. All statistical analyses were performed with the SPSS statistical software program (version 29.0 of Mac OS, SPSS Inc., Chicago, IL, U.S.A.). We performed a probit analysis of the data using MINITAB version 17.1 and the maximum likelihood method to evaluate the distribution fit of the data and the significant effects of ES2 as ovicidal and larvicidal activities. A scheme of the research steps is illustrated (Fig. 3).

### Supplementary Information

Supplementary Information 1.

Supplementary Information 2.

## Data Availability

The datasets generated during and/or analyzed during the current study are available from the corresponding author on reasonable request.

## Abbreviations

DEHP: Di (2-ethylhexyl) phthalate
DW: Dry weight
EC number: Enzyme commission number
ES2: Endophytic *Streptomyces* studied species
EU: Enzyme Unit
*F*.: Family
GABA: γ-Aminobutyric acid
HPLC: High-performance liquid chromatography
LC: Liquid chromatography
LC-QTOF-MS/MS: Liquid chromatography combined with quadrupole-time-of-flight high-definition mass spectrometry
m/zvalue: Mass divided by charge number and detected by mass spectrometry
M + H: Molecule + hydrogen atom
p-value: Probability under the assumption of no effect or no difference
R. H.: Relative humidity
*S*.: *Spodoptera*
SC: Suspension concentrate
*St*.: *Streptomyces*
TiO_2_: Titanium dioxide
