# Supplementary material for: Metabolite profiling and in-silico studies show multiple effects of insecticidal actinobacterium on Spodoptera littoralis
Source: Sci Rep. 2024 Feb 6;14:3057. doi: 10.1038/s41598-024-53096-y (PMC10847143; doi:10.1038/s41598-024-53096-y)
Supplement: Supplementary file 1 — Supplementary Information 1. [file 41598_2024_53096_MOESM1_ESM.pdf]

Article Title

Metabolite Profiling and In-Silico Studies Show Multiple Effects of Insecticidal Actinobacterium on *Spodoptera littoralis*

Authors

Mohamed Diab[1*], Hala Mead[1], Mohamad Khedr[2], Mohamed S. Nafie[3], Abdelghafar Abu-Elsaoud[4], Sahar El-Shatoury[4]

Affiliations

1. Agricultural Research Center, Plant Protection Research Institute, Pest Physiology Department, 12311 Giza, Egypt

2. Agricultural Research Center, Plant Protection Research Institute, Cotton Leafworm Department, 12311 Giza, Egypt

3. Suez Canal University, Faculty of Science, Chemistry Department, 41522 Ismailia, Egypt

4. Suez Canal University, Faculty of Science, Microbiology & Botany Department, 41522 Ismailia, Egypt

***Mohamed Khaled Diab:** Agricultural Research Center, Plant Protection Research Institute, Pest Physiology Department, 12311 Giza, Egypt; mohamed.diab_pgs@science.suez.edu.eg; orcid.org/0000-0001-7879-1357

Hala Mohamed Mead: Agricultural Research Center, Plant Protection Research Institute, Pest Physiology Department, 12311 Giza, Egypt; hmimead@yahoo.com; orcid.org/0000-0002-5625-7281

Mohamad Ahmad Khedr: Agricultural Research Center, Plant Protection Research Institute, Cotton Leafworm Department, 12311 Giza, Egypt; m1khedr@yahoo.com; orcid.org/0000-0001-8549-1336

Mohamed S. Nafie: Suez Canal University, Faculty of Science, Chemistry Department, 41522 Ismailia, Egypt; mohamed_nafie@science.suez.edu.eg; orcid.org/0000-0003-4454-6390

Abdelghafar Mohamed Abu-Elsaoud: Suez Canal University, Faculty of Science, Botany & Microbiology Department, 41522 Ismailia, Egypt; abuelsaoud@science.suez.edu.eg; orcid.org/0000-0002-6269-3418

Sahar Ahmed El-Shatoury: Suez Canal University, Faculty of Science, Botany & Microbiology Department, 41522 Ismailia, Egypt; sahar_hassan@science.suez.edu.eg; orcid.org/0000-0002-6093-5145

Corresponding author

[Mohamed Diab: (+201000087158) (mohamed.diab_pgs@science.suez.edu.eg)]

Supplementary information

Supplementary Table S1. Completely identified compounds in the ethyl acetate extract of *Streptomyces* ES2 EMCC2291 by LC-QTOF-MS/MS.

Supplementary Table S1. Completely identified compounds in the ethyl acetate extract of *Streptomyces* ES2 EMCC2291 by LC-QTOF-MS/MS.

No.	RT (min.)	Intensity	Mass (Da)	Adduct	m/z value (mass)*	Molecular formula	Identified compound	InChI Key	Chemical structure	Chemical class	ChemSpider ID*, KEGG ID**, PubChem CID***, METLIN ID****, CAS Registry Number#
1	0.4823	3067.855	221.64	$[M+H]^+$	222.062	$C_{11}H_8ClNO_2$	Quinmerac	ALZOLUNSQWINIR-UHFFFAOYSA-N	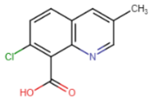	Acid	82847*, C18891**, 91749***, NA****, 90717-03-6#
2	0.4948	29738.15	183.163	$[M+H]^+$	184.1085	$C_8H_9NO_4$	4-Pyridoxate	HXACOUQIXZGNBF-UHFFFAOYSA-N	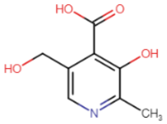	Acid	6467*, C00847**, 6723***, 239****, 82-82-6#
3	0.5087	24269.63	95.101	$[M+H]^+$	96.04395	C_5H_5NO	3-Hydroxypyridine	GRFNBEZIAWKNCO-UHFFFAOYSA-N	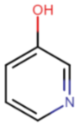	Phenol	7683*, NA**, 7971***, NA****, 109-00-2#
4	0.5087	38747.27	139.11	$[M+H]^+$	140.0703	$C_6H_5NO_3$	6-Hydroxynicotinate	BLHCMGRVFXRYRN-UHFFFAOYSA-N	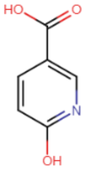	Acid	65756*, C01020**, 72924***, NA****, 5006-66-6#
5	0.5221	38079.43	135.1267	$[M+H]^+$	136.0603	$C_5H_5N_5$	Adenine	GFFGJBXGBJISGV-UHFFFAOYSA-N	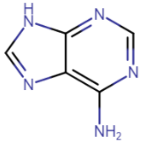	Aromatic amine (Nitrogenous base)	185*, C00147**, 190***, 85****, 73-24-5#

Supplementary Table S1. Continued.

No.	RT (min.)	Intensity	Mass (Da)	Adduct	m/z value (mass)*	Molecular formula	Identified compound	InChI Key	Chemical structure	Chemical class	ChemSpider ID*, KEGG ID**, PubChem CID***, METLIN ID****, CAS Registry Number#
6	0.5221	31600.33	169.1778	$[M+H]^+$	170.0822	$C_8H_{11}NO_3$	Pyridoxine	LXNHXLLTXMVWPM-UHFFFAOYSA-N	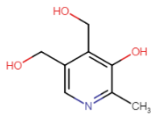	Phenol	1025*, C00314**, 1054***, NA****, 65-23-6#
7	0.5400	2728	137.0841	$[M+H]^+$	138.09134	$C_8H_{11}NO$	Tyramine	DZGWFCGJZKJUFP-UHFFFAOYSA-N	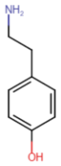	Phenol	5408*, C00483**, 5610***, 60****, 51-67-2#
8	0.5400	23538	277.1175	$[M+H]^+$	278.12477	$C_{12}H_{15}N_5O_3$	Queuine	WYROLENTHWJFLR-ACLDMZEESA-N	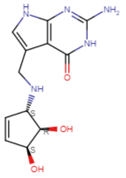	Aromatic amine (Nitrogenous base)	102837*, C01449**, 135398670***, 6279****, 86496-18-6#
9	0.5479	21011.8	115.217	$[M+H]^+$	116.07	$C_7H_{17}N$	Butylisopropylamine	OKRJGUKZYSEUOY-UHFFFAOYSA-N	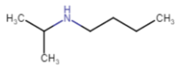	Aliphatic hydrocarbon	110075*, NA**, 123480***, NA****, 7515-80-2#
10	0.5600	8194	165.0789	$[M+H]^+$	166.08626	$C_9H_{11}NO_2$	L-Phenylalanine	COLNVLDHVKWLRT-QMMMGPOBSA-N	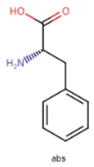	Acid	5910*, C00079**, 6140***, 28****, 63-91-2#

Supplementary Table S1. Continued.

No.	RT (min.)	Intensity	Mass (Da)	Adduct	m/z value (mass)*	Molecular formula	Identified compound	InChI Key	Chemical structure	Chemical class	ChemSpider ID*, KEGG ID**, PubChem CID***, METLIN ID****, CAS Registry Number#
11	0.5700	4249	169.0739	$[M+H]^+$	170.08117	$C_8H_{11}NO_3$	Pyridoxine	LXNHXLLTXMVWPM-UHFFFAOYSA-N	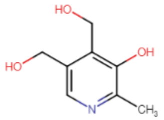	Phenol	1025*, C00314**, 1054***, 5245****, 65-23-6#
12	0.5800	1066	167.0582	$[M+H]^+$	168.06552	$C_8H_9NO_3$	Pyridoxal	RADKZDMFGJYCBB-UHFFFAOYSA-N	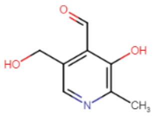	Phenol	1021*, C00250**, 1050***, 6312****, 63907-53-9#
13	0.6000	817	122.048	$[M+H]^+$	123.05529	$C_6H_6N_2O$	Niacinamide	DFPAKSUCGFBDDF-UHFFFAOYSA-N	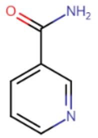	Amide (1◦)	911*, C00153**, 936***, 1497****, 98-92-0#
14	0.6200	835	126.0429	$[M+H]^+$	127.0502	$C_5H_6N_2O_2$	Thymine	RWQNBRDOKXIBIV-UHFFFAOYSA-N	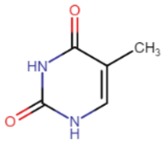	Amide (2◦)	1103*, C00178**, 1135***, 290****, 65-71-4#
15	0.6300	51937	163.0858	$[M+H]^+$	164.09307	$C_7H_9N_5$	9-ethylpurin-6-amine	MUIPLRMGAXZWSQ-UHFFFAOYSA-N	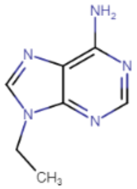	Aromatic amine	6*, NA**, 7***, NA****, 2715-68-6#

Supplementary Table S1. Continued.

No.	RT (min.)	Intensity	Mass (Da)	Adduct	m/z value (mass)*	Molecular formula	Identified compound	InChI Key	Chemical structure	Chemical class	ChemSpider ID*, KEGG ID**, PubChem CID***, METLIN ID****, CAS Registry Number#
16	0.6409	39897.31	159.0199	$[M+H]^+$	160.0747	$C_5H_6ClN_3O$	Chloridazon-methyl-desphenyl	XNSGCNYTNLWRKM-UHFFFAOYSA-N	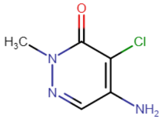	Amide (3◦)	516658*, NA**, 594330***, NA****, NA#
17	0.6541	24586.95	175.0481	$[M+H]^+$	176.0919	$C_6H_9NO_5$	N-Acetyl-DL-aspartic acid	OTCCIMWXFLJLIA-UHFFFAOYSA-N	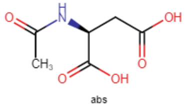	Acid	88007*, NA**, 97508***, NA****, 2545-40-6#
18	0.6900	776	148.0736	$[M+H]^+$	149.08084	$C_6H_{12}O_4$	(2R,3R)-2,3-dihydroxy-3-methyl pentanoic acid	PDGXJDXVGMHUIR-UHFFFAOYSA-N	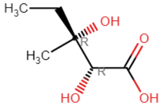	Acid	395044*, C06007**, 448154***, NA****, NA#
19	0.7150	70192.07	99.10479	$[M+H]^+$	100.0749	$C_6H_{11}NH_2$ or $C_6H_{13}N$	Cyclohexanamine	PAFZNILMFXTMIY-UHFFFAOYSA-N	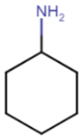	Aliphatic amine (1◦)	7677*, C00571**, 7965***, NA****, 157973-60-9#
20	0.7316	14100.1	128.0949	$[M+H]^+$	129.1022	$C_6H_{12}N_2O$	4-Piperidinecarboxamide	DPBWFNDFMCCGGJ-UHFFFAOYSA-N	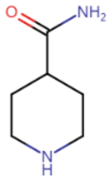	Amide (1◦)	3640*, NA44**, 3772***, NA****, 39546-32-2#

Supplementary Table S1. Continued.

No.	RT (min.)	Intensity	Mass (Da)	Adduct	m/z value (mass)*	Molecular formula	Identified compound	InChI Key	Chemical structure	Chemical class	ChemSpider ID*, KEGG ID**, PubChem CID***, METLIN ID****, CAS Registry Number#
21	0.7449	8658.977	143.0405	$[M+H]^+$	144.047	C_6H_9NOS	4-Methyl-5-thiazole-ethanol	BKAWJIRCKVUVED-UHFFFAOYSA-N	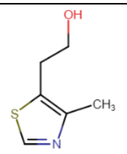	Aromatic alcohol	1104*, C04294**, 1136***, NA****, 137-00-8#
22	0.7700	956	143.0405	$[M+H]^+$	144.04776	C_6H_9NOS	5-(2-Hydroxyethyl)-4-methyl thiazole	BKAWJIRCKVUVED-UHFFFAOYSA-N	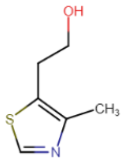	Aromatic alcohol	1104*, C04294**, 1136***, NA****, 137-00-8#
23	0.7969	33728.8	151.1361	$[M+H]^+$	152.1275	$C_{10}H_{17}N$	Amantadine	DKNWSYNQZKUICI-UHFFFAOYSA-N	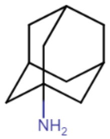	Aliphatic amine (1◦)	2045*, C06818**, 2130***, NA****, 768-94-5#
24	0.8096	207353.1	134.0943	$[M+H]^+$	135.1004	$C_6H_{14}O_3$ or $(CH_3OCH_2CH_2)_2O$	Diglyme	SBZXBUIDTXKZTM-UHFFFAOYSA-N	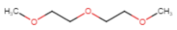	Aliphatic ether	13839575*, NA**, 8150***, NA****, 111-96-6#
25	0.9714	12436.39	139.1088	$[M+H]^+$	140.1089	$C_6H_5NO_3$	4-Nitrophenol	BTJIUGUIPKRLHP-UHFFFAOYSA-N	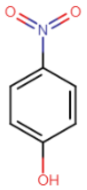	Phenol	955*, C00870**, 980***, 4100****, 100-02-7#

Supplementary Table S1. Continued.

No.	RT (min.)	Intensity	Mass (Da)	Adduct	m/z value (mass)*	Molecular formula	Identified compound	InChI Key	Chemical structure	Chemical class	ChemSpider ID*, KEGG ID**, PubChem CID***, METLIN ID****, CAS Registry Number#
26	1.0969	28438.13	181.1885	$[M+H]^+$	182.1177	$C_9H_{11}NO_3$	L-Tyrosine	OUYCCCASQSFEME-QMMMGPOBSA-N	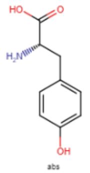	Amino acid	5833*, C00082**, 6057***, 34****, 60-18-4#
27	1.5514	1946444	115.217	$[M+H]^+$	116.1066	$C_7H_{17}N$	N-tert-Butylisopropylamine	ZWXQPERWRDHCMZ-UHFFFAOYSA-N	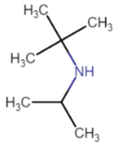	Amine (2◦)	74027*, NA**, 82023***, NA****, 7515-80-2#
28	2.0632	30407.36	267.1834	$[M+H]^+$	268.1674	$C_{15}H_{25}NO_3$	Metoprolol	IUBSYMUCCVWXPE-UHFFFAOYSA-N	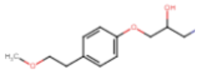	Alcohol	4027*, C07202**, 4171***, 1250****, 29122-76-7#
29	2.0888	21054.61	147.0684	$[M+H]^+$	148.0743	C_9H_9NO	3-Methyloxyindole	BBZCPUCZKLTAJQ-UHFFFAOYSA-N	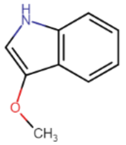	Aromatic amine	133020*, C02366**, 150923***, NA****, 339280-26-1#
30	2.1392	85513.11	117.1463	$[M+H]^+$	118.1222	$C_5H_{11}NO_2$	Betaine	KWIUHFFTVRNATP-UHFFFAOYSA-N	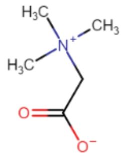	Amine (Quaternary)	242*, C00719**, 247***, 287****, 105371-61-7#

Supplementary Table S1. Continued.

No.	RT (min.)	Intensity	Mass (Da)	Adduct	m/z value (mass)*	Molecular formula	Identified compound	InChI Key	Chemical structure	Chemical class	ChemSpider ID*, KEGG ID**, PubChem CID***, METLIN ID****, CAS Registry Number#
31	2.7052	10400.56	133.151	$[M+H]^{+}$	134.0585	$C_7H_7N_3$	5-Methyl-1H-benzotriazole	LRUDIIUSNGCQKF-UHFFFAOYSA-N	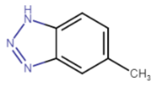	Aromatic amine	8381*, NA**, 8705***, NA****, 49636-02-4#
32	3.2751	7885.926	203.1158	$[M+H]^{+}$	204.1253	$C_9H_{17}NO_4$	O-Acetyl-L-carnitine	RDHQFKQIGNGIED-MRVPVSSYSA-O	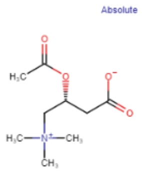	Amine (Quaternary)	5406074*, NA**, 7045767***, NA****, 3040-38-8#
33	3.4676	56928.55	166.0967	$[M+H]^{+}$	167.1178	$C_6H_{10}N_6$	Cyromazine	LVQDKIWDGQRHTE-UHFFFAOYSA-N	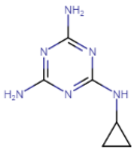	Aromatic amine	43550*, C14147**, 47866***, NA****, 66215-27-8#
34	3.6500	979	331.1332	$[M+H]^{+}$	332.1405	$C_{17}H_{18}FN_3O_3$	Ciprofloxacin	MYSWGUAQZAJSOK-UHFFFAOYSA-N	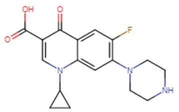	Acid	2662*, C05349**, 2764***, NA****, 438571-52-9#
35	3.8700	4201	149.0702	$[M+H]^{+}$	150.07742	$C_6H_7N_5$	3-Methyladenine	ZPBYVFQJHWLTFB-UHFFFAOYSA-N	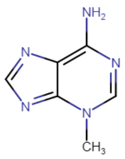	Aromatic amine (Nitrogenous base)	1610*, C00913**, 135398661***, NA****, 5142-23-4#

Supplementary Table S1. Continued.

No.	RT (min.)	Intensity	Mass (Da)	Adduct	m/z value (mass)*	Molecular formula	Identified compound	InChI Key	Chemical structure	Chemical class	ChemSpider ID*, KEGG ID**, PubChem CID***, METLIN ID****, CAS Registry Number#
36	3.8957	21674.79	149.0511	$[M+H]^+$	150.0759	$C_5H_{11}NO_2S$	Methionine	FFEARJCKVFRZRR-UHFFFAOYSA-N	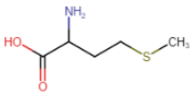	Amino acid	5907*, C00073**, 6137***, 5664****, 59-51-8#
37	4.0231	19367.84	121.0892	$[M+H]^+$	122.0959	$C_8H_{11}N$	2,6-Xylidine	UFFBMTHBGFGIHF-UHFFFAOYSA-N	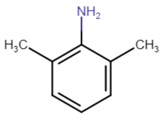	Amide (1◦)	6630*, C11004**, 6896***, NA****, 21436-98-6#
38	4.0231	77272.77	163.0997	$[M+H]^+$	164.1066	$C_{10}H_{13}NO$	N-(2-phenylethyl) acetamide	MODKMHXGCGKTLE-UHFFFAOYSA-N	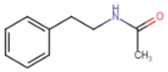	Ketone	63331*, C06746**, 70143***, NA****, 877-95-2#
39	4.2100	31674	283.1321		283.13208	$C_{16}H_{17}N_3O_2$	Brevianamid F	RYFZBPVMVYTEKZ-KBPBESRZSA-N	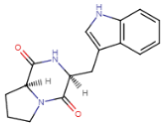	Amide (2◦)	157941*, C20563**, 181567***, NA****, 38136-70-8#
40	4.4405	9243.612	219.0752	$[M+H]^+$	220.137	$C_9H_{17}NOS_2$	1-Isothiocyanato-7-(methylsulfinyl)-heptane	OGYHCBGORZWBPH-UHFFFAOYSA-N	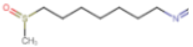	Sulfur containing compound	8013088*, NA**, 9837367***, NA****, NA#

Supplementary Table S1. Continued.

No.	RT (min.)	Intensity	Mass (Da)	Adduct	m/z value (mass)*	Molecular formula	Identified compound	InChI Key	Chemical structure	Chemical class	ChemSpider ID*, KEGG ID**, PubChem CID***, METLIN ID****, CAS Registry Number#
41	4.5300	10605.71	143.0735	$[M+H]^+$	144.0807	$C_{10}H_9N$	1-Naphthylamine	RUFPHBVGCFYCNW-UHFFFAOYSA-N	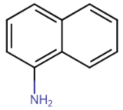	Aromatic amine	8319*, C14790**, 8640***, NA****, 134-32-7#
42	4.5600	7005	150.0681	$[M+H]^+$	151.07536	$C_9H_{10}O_2$	4-Vinylguaiacol	YOMSJEATGXXYPX-UHFFFAOYSA-N	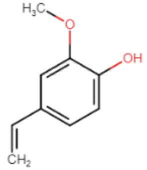	Phenol	325*, C17883**, 332***, NA****, 112602-32-1#
43	4.6062	57970.63	146.0368	$[M+H]^+$	147.0797	$C_9H_6O_2$	Coumarin	ZYGHJZDHTFUPRJ-UHFFFAOYSA-N	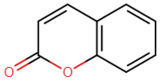	α ,β unsaturated carbonyl compound	13848793*, C05851**, 323***, 3525****, 91-64-5#
44	4.6062	12078.19	330.2406	$[M+H]^+$	353.1287	$C_{18}H_{34}O_5$	(Z)-9,12,13-trihydroxyoctadec-15-enoic acid	DNWUYCUUEGGVPR-CLTKARDFSA-N	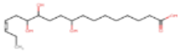	Acid	23550999*, C14833**, 24066906***, NA****, NA#
45	5.0583	9966.364	190.0629	$[M+H]^+$	191.0706	$C_{11}H_{10}O_3$	Hymecromone Methyl Ether	UDFPKNSWSYBIHO-UHFFFAOYSA-N	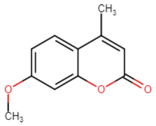	α, β unsaturated carbonyl compound	346415*, NA**, 390807***, NA****, NA#

Supplementary Table S1. Continued.

No.	RT (min.)	Intensity	Mass (Da)	Adduct	m/z value (mass)*	Molecular formula	Identified compound	InChI Key	Chemical structure	Chemical class	ChemSpider ID*, KEGG ID**, PubChem CID***, METLIN ID****, CAS Registry Number#
46	5.5906	5178.966	165.0789	$[M+H]^+$	166.0843	$C_9H_{11}NO_2$	Phenylalanine	COLNVLDHVKWLRT-UHFFFAOYSA-N	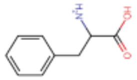	Amino acid	969*, C00079**, 994***, 28****, 150-30-1#
47	5.8365	19292.65	236.1776	$[M+H]^+$	237.1487	$C_{15}H_{24}O_2$	2-hexyl-5-propyl-resorcinol	VERGPVBZPMTZDY-UHFFFAOYSA-N	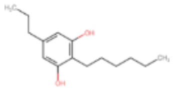	Phenol	170764*, NA**, 197183***, NA****, 39341-78-1#
48	5.8621	6086.6	231.1561	$[M+H]^+$	232.1561	NA	2-[(2-hydroxy-3-methyl butanol) amino]-4-methyl pentanoic acid	SWEPQJJUAVEABL-UHFFFAOYSA-N	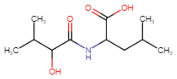	Acid	NA*, NA**, NA***, NA****, 140681-91-0#
49	5.8900	43400	280.1311		280.13107	$C_{15}H_{20}O_5$	Heptelidic acid	JESMSCGUTIEROV-UHFFFAOYSA-N	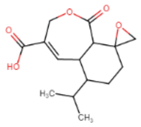	Acid	9121060*, NA**, 10016502***, NA****, 57710-57-3#
50	6.3135	5132.789	390.277	$[M+H]^+$	391.1884	$C_{24}H_{38}O_4$	Di(2-ethylhexyl) phthalate (DEHP)	BJQHLKABXJIVAM-UHFFFAOYSA-N	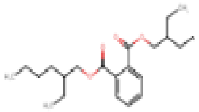	Ester	21106505*, C03690**, 8343***, NA****, 117-81-7#

Supplementary Table S1. Continued.

No.	RT (min.)	Intensity	Mass (Da)	Adduct	m/z value (mass)*	Molecular formula	Identified compound	InChI Key	Chemical structure	Chemical class	ChemSpider ID*, KEGG ID**, PubChem CID***, METLIN ID****, CAS Registry Number#
51	6.3476	13737.77	289.1678	$[M+Na]^+$	312.1266	$C_{17}H_{23}NO_3$	Atropine	RKUNBYITZUJHSG-PJPHBNEVSA-N	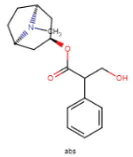	Ester	10194105*, C01479**, 174174***, NA****, 803615-91-0#
52	6.6353	15188.73	276.1209	$[M+H]^+$	277.1297	$C_{12}H_{20}O_7$	Triethylcitrate	DOOTYTYQINUNNV-UHFFFAOYSA-N	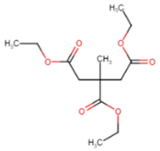	Ester	13850879*, D06228**, 6506***, NA****, 77-93-0#
53	6.6700	11065	341.2566	$[M+H]^+$	342.26389	$C_{19}H_{35}NO_4$	Dodecenoylcarnitine	VFYGOZNDDVMNNT-UHFFFAOYSA-N	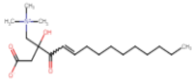	Aliphatic hydrocarbon	30776709*, NA**, 129664620***, NA****, NA#
54	6.8179	14005.92	210.1256	$[M+H]^+$	211.1690	$C_{12}H_{18}O_3$	Jasmonic Acid	ZNJFBWYDHIGLCU-UHFFFAOYSA-N	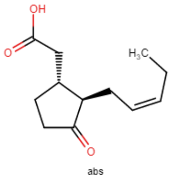	Acid	4444606*, C08491**, 5281166***, NA****, 221682-41-3#
55	7.3946	27072.88	178.0266	$[M+H]^+$	179.1438	$C_9H_6O_4$	esculetin	ILEDWLMCKZNDJK-UHFFFAOYSA-N	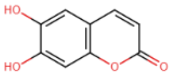	Phenol	4444764*, C09263**, 5281416***, NA****, 305-01-1#

Supplementary Table S1. Continued.

No.	RT (min.)	Intensity	Mass (Da)	Adduct	m/z value (mass)*	Molecular formula	Identified compound	InChI Key	Chemical structure	Chemical class	ChemSpider ID*, KEGG ID**, PubChem CID***, METLIN ID****, CAS Registry Number#
56	7.4374	17387.97	196.0596	$[M+H]^+$	197.1155	$C_7H_8N_4O_3$	1,9-dimethyl uric acid	UARKDOLETOEBCU-UHFFFAOYSA-N	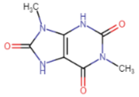	Amide (3◦)	97753*, C16356**, 108712***, NA****, 55441-62-8#
57	7.7400	23333	356.1631	$[M+H]^+$	357.17034	$C_{14}H_{24}N_6O_3S$	S-adenosylmethioninaminium	ZUNBITIXDCPNSD-LSRJEVITSA-O	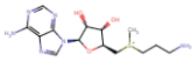	Aromatic amine (Nitrogenous base)	26330790*, EC 2.5.1.6**, 25203490***, NA****, NA#
58	7.8800	6899	484.0772	$[M+H]^+$	485.08452	$C_{21}H_{21}ClO_{11}$	Cyanidin 3-O-glucoside	YTMNONATNXDQJF-UBNZBFALSA-N	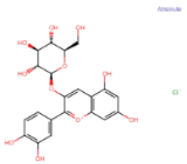	Phenol	170681*, NA**, 197081***, NA****, 47705-70-4#
59	8.9500	99655	254.1518	$[M+H]^+$	255.15909	$C_{14}H_{22}O_4$	Palitantin	MPOXQBRZHHNMER-XZQMCIKJSA-N	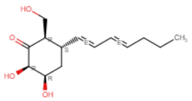	Phenol	4942905*, NA**, 6438427***, NA****, 15265-28-8#
60	9.2096	1023536	148.0160	$[M+H]^+$	149.0227	$C_8H_4O_3$	Phthalic anhydride	LGRFSURHDFAFJT-UHFFFAOYSA-N	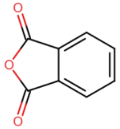	Anhydride	6552*, NA**, 6811***, NA****, 85-44-9#

Supplementary Table S1. Continued.

No.	RT (min.)	Intensity	Mass (Da)	Adduct	m/z value (mass)*	Molecular formula	Identified compound	InChI Key	Chemical structure	Chemical class	ChemSpider ID*, KEGG ID**, PubChem CID***, METLIN ID****, CAS Registry Number#
61	9.2000	165301	176.0473	$[M+H]^+$	177.05462	$C_{10}H_8O_3$	4-methylumbelliferone	HSHNITRMYYLLCV-UHFFFAOYSA-N	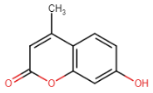	α, β unsaturated carbonyl compound	4444190*, C03081**, 5280567***, NA****, 90-33-5#
62	9.2800	34217	312.1573	$[M+H]^+$	313.16457	$C_{16}H_{24}O_6$	Pyrenophorol	RBQNDQOKFICJGL-WZJCORALSA-N	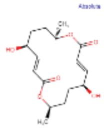	Alcohol	9111106*, NA**, 10935870***, NA****, 22248-41-5#
63	9.4564	24186.26	266.1518	$[M+Na]^+$	289.1995	$C_{15}H_{22}O_4$	2,8-Dihydroxy-5,5,8-trimethyl-11-oxatetracyclo [7.3.1.01,9.03,7] tridecan-10-one	VXHVLJHZFYIFJE-QIEUJACUSA-N	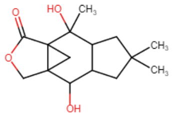	Alcohol	291811*, NA**, 329432***, NA****, NA#
64	9.4500	7017	326.1453	$[M+H]^+$	327.15256	$C_{19}H_{22}N_2OS$	Acepromazine	NOSIYYJFMPDDSA-UHFFFAOYSA-N	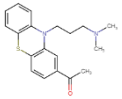	Ketone	5852*, D07065**, 6077***, NA****, 61-00-7#
65	9.6600	6665	418.2719	$[M+H]^+$	419.2792	$C_{25}H_{38}O_5$	Simvastatin	RYMZZMVNJRMUDD-HGQWONQESA-N	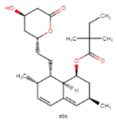	Ester	49179*, D00434**, 54454***, 2443****, 79902-39-9#

Supplementary Table S1. Continued.

No.	RT (min.)	Intensity	Mass (Da)	Adduct	m/z value (mass)*	Molecular formula	Identified compound	InChI Key	Chemical structure	Chemical class	ChemSpider ID*, KEGG ID**, PubChem CID***, METLIN ID****, CAS Registry Number#
66	9.6604	102507.2	416.1682	$[M+Na]^+$	439.2093	$C_{19}H_{28}O_{10}$	beta-D-Glucopyranoside, 2-phenylethyl 6-O-beta-D-xylopyranosyl-	ZRGXCWYRIBRSQA-UHFFFAOYSA-N	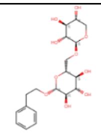	Phenol	115929*, NA**, 14704521***, NA****, NA#
67	9.7509	17524.42	318.2195	$[M+Na]^+$	341.1791	$C_{20}H_{30}O_3$	1-Phenanthrene carboxylic acid, 7-ethenyl-1,2,3,4,4a,4b,5,6,7,9,10,10a-dodecahydro-9-hydroxy-1,4a,7-trimethyl-	PLKOJNUQWAKPNC-UHFFFAOYSA-N	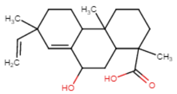	Acid	29814271*, NA**, 14864259***, NA****, NA#
68	10.160	12259	278.1267	$[M+H]^+$	279.13393	$C_{14}H_{18}N_2O_4$	Oxadixyl	UWVQIROCRJWDKL-UHFFFAOYSA-N	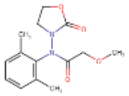	Ester	48518*, C18753**, 53735***, NA****, 77732-09-3#
69	10.180	49767	248.1412	$[M+H]^+$	249.14852	$C_{15}H_{20}O_3$	enokipodins C	OXZGFGFICRZIFY-DURCKFPESA-N	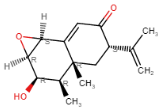	α, β unsaturated carbonyl compound	35467340*, NA**, 101316756***, NA****, 28619-41-2#

Supplementary Table S1. Continued.

No.	RT (min.)	Intensity	Mass (Da)	Adduct	m/z value (mass)*	Molecular formula	Identified compound	InChI Key	Chemical structure	Chemical class	ChemSpider ID*, KEGG ID**, PubChem CID***, METLIN ID****, CAS Registry Number#
70	10.183	142468	266.337	[M-H2O +H]$^+$	249.1492	$C_{15}H_{22}O_4$	3-hydroxy-4-(2-hydroxy-6-methylheptan-2-yl) benzoic acid	VZXPWVDKXC YHSI-HNNXBMFYSA-N	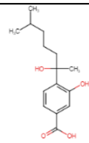	Acid	NA*, NA**, 25775004***, NA****, NA#
71	10.290	2299	458.1788	[M+H]$^+$	459.18609	$C_{21}H_{30}O_{11}$	Deoxynivalenol-3-glucoside	PUMXWMGECQ IOGB-SMSDQXDJSA-N	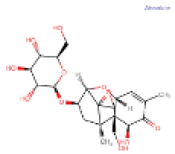	Phenol	34991041*, NA**, 71312510***, NA****, 131180-21-7#
72	10.680	17542	415.2723	[M+H]$^+$	416.27954	$C_{25}H_{37}NO_4$	Salmeterol	GIIZNNXWQWC KIB-UHFFFAOYSA-N	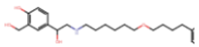	Phenol	4968*, C07241**, 5152***, NA****, 840530-57-6#
73	11.020	6871	166.0967	[M+H]$^+$	167.10397	$C_6H_{10}N_6$	Cyromazine	LVQDKIWDGQR HTE-UHFFFAOYSA-N	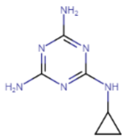	Aromatic amine	43550*, C14147**, 47866***, NA****, 66215-27-8#
74	12.420	2902	267.0968	[M+H]$^+$	268.10403	$C_{10}H_{13}N_5O_4$	2-deoxyguanosine	YKBGVTZYEHR EMT-KVQBGUIXSA-N	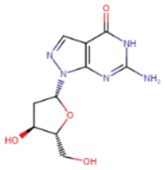	Aromatic amine (Nitrogenous base)	163230*, C00330**, 135398592***, 3395****, 79971-08-7#

Supplementary Table S1. Continued.

No.	RT (min.)	Intensity	Mass (Da)	Adduct	m/z value (mass)*	Molecular formula	Identified compound	InChI Key	Chemical structure	Chemical class	ChemSpider ID*, KEGG ID**, PubChem CID***, METLIN ID****, CAS Registry Number#
75	13.020	1941	304.1011	$[M+H]^+$	305.10833	$C_{12}H_{21}N_2O_3PS$	Diazinon	FHIVAFMUCKRCQO-UHFFFAOYSA-N	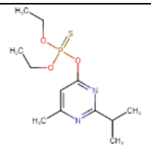	Aromatic amine	2909*, C14324**, 3017***, NA****, 333-41-5#
76	13.610	3625	164.0837	$[M+H]^+$	165.09101	$C_{10}H_{12}O_2$	4-Allyl-2-methoxyphenol "Eugenol"	RRAFCDWBNXTKKO-UHFFFAOYSA-N	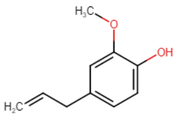	Phenol	13876103*, C10453**, 3314***, 4022****, 97-53-0#
77	14.348	92873.76	272.1776	$[M+H]^+$	273.1874	$C_{18}H_{24}O_2$	Galaxolidone	PGMHPYRIXBRRQD-UHFFFAOYSA-N	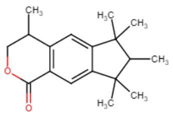	α, β unsaturated carbonyl compound	28290252*, NA**, 69131857***, NA****, NA#
78	16.754	41945.72	330.1831	$[M+H]^+$	331.1911	$C_{20}H_{26}O_4$	Dicyclohexyl phthalate	VOWAEIGWURALJQ-UHFFFAOYSA-N	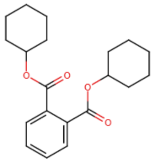	Ester	6519*, C14529**, 6777***, NA****, 84-61-7#
79	16.822	37264.16	250.0395	$[M+H]^+$	251.0474	$C_{12}H_{11}O_4P$	Diphenyl phosphate	ASMQGLCHMVWBQR-UHFFFAOYSA-N	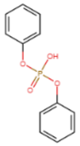	Phosphorous compound	12722*, NA**, 13282***, NA****, 48168-03-2#

Supplementary Table S1. Continued.

No.	RT (min.)	Intensity	Mass (Da)	Adduct	m/z value (mass)*	Molecular formula	Identified compound	InChI Key	Chemical structure	Chemical class	ChemSpider ID*, KEGG ID**, PubChem CID***, METLIN ID****, CAS Registry Number#
80	17.450	1559	412.3705	$[M+H]^+$	413.37779	$C_{29}H_{48}O$	14-demethyllanosterol	CHGIKSSZNBCNDW-GKBRUXRCSA-N	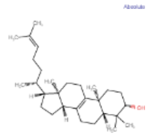	Phenol	145139*, C05108**, 50990081***, 6135****, 7448-02-4#
81	19.790	11626	790.5231	$[M+H]^+$	791.53039	$C_{45}H_{74}O_{11}$	Oligomycin A	MNULEGDCPYONBU-WMBHJXFZSA-N	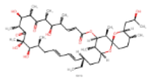	Phenol	10308285*, C11311**, 5281899***, NA****, 14104-19-9#
82	21.423	1228978	390.277	$[M+H]^+$	391.283	$C_{24}H_{38}O_4$	Dioctyl Phthalate	MQIUGAXCHLFZKX-UHFFFAOYSA-N	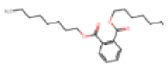	Ester	8043*, C03690**, 8346***, NA****, 68515-43-5#
83	23.100	6099	367.2259	$[M+H]^+$	368.22598	$C_{22}H_{29}N_3O_2$	Brevicompanine B	HAXPBJUEOMQIJN-UHFFFAOYSA-N	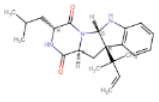	Diketone	8960513*, NA**, 51340320***, NA****, 215121-47-4#

* refers to ChemSpider ID; ** refers to KEGG ID; *** refers to PubChem CID; **** refers to METLIN ID; # refers to CAS Registry Number

Mass (Da); molecular weight in Dalton. (m/z) are values detected by mass spectrometry; RT, retention time; NA, not available.

Chemical structures were carried out using Reaxys ChemDraw software, version 18.0.0.20 (https://www.reaxys.com.mplbci.ekb.eg/chemdrawservices/restn).

LC-QTOF-MS/MS, liquid chromatography, combined with quadrupole-time-of-flight high-definition mass spectrometry instrument.

CAS Registry Number, Chemical Abstracts Service database (https://www.cas.org/); ChemSpider ID, Chemical structure Database Identifier (http://www.chemspider.com/); Errors (ppm) were obtained by formula prediction software in the mass spectrometer; InChI Key, International Chemical Identifier (https://pubchem.ncbi.nlm.nih.gov/source/ChEBI); KEGG ID, Kyoto Encyclopedia of Genes and Genomes (http://www.genome.jp/kegg); METLIN ID, METLIN Metabolite and Chemical Entity Database (https://metlin.scripps.edu/); PubChem CID, A Database of Chemical Molecules and Their Activities against Biological Assays (https://pubchem.ncbi.nlm.nih.gov/).
