# Supplementary material for: Metabolite profiling and in-silico studies show multiple effects of insecticidal actinobacterium on Spodoptera littoralis
Source: Sci Rep. 2024 Feb 6;14:3057. doi: 10.1038/s41598-024-53096-y (PMC10847143; doi:10.1038/s41598-024-53096-y)
Supplement: Supplementary file 2 — Supplementary Information 2. [file 41598_2024_53096_MOESM2_ESM.pdf]

Article Title

Metabolite Profiling and In-Silico Studies Show Multiple Effects of Insecticidal Actinobacterium on *Spodoptera littoralis*

Authors

Mohamed Diab[1*], Hala Mead[1], Mohamad Khedr[2], Mohamed S. Nafie[3], Abdelghafar Abu-Elsaoud[4], Sahar El-Shatoury[4]

Affiliations

1. Agricultural Research Center, Plant Protection Research Institute, Pest Physiology Department, 12311 Giza, Egypt

2. Agricultural Research Center, Plant Protection Research Institute, Cotton Leafworm Department, 12311 Giza, Egypt

3. Suez Canal University, Faculty of Science, Chemistry Department, 41522 Ismailia, Egypt

4. Suez Canal University, Faculty of Science, Microbiology & Botany Department, 41522 Ismailia, Egypt

***Mohamed Khaled Diab:** Agricultural Research Center, Plant Protection Research Institute, Pest Physiology Department, 12311 Giza, Egypt; mohamed.diab_pgs@science.suez.edu.eg; orcid.org/0000-0001-7879-1357

Hala Mohamed Mead: Agricultural Research Center, Plant Protection Research Institute, Pest Physiology Department, 12311 Giza, Egypt; hmimead@yahoo.com; orcid.org/0000-0002-5625-7281

Mohamad Ahmad Khedr: Agricultural Research Center, Plant Protection Research Institute, Cotton Leafworm Department, 12311 Giza, Egypt; m1khedr@yahoo.com; orcid.org/0000-0001-8549-1336

Mohamed S. Nafie: Suez Canal University, Faculty of Science, Chemistry Department, 41522 Ismailia, Egypt; mohamed_nafie@science.suez.edu.eg; orcid.org/0000-0003-4454-6390

Abdelghafar Mohamed Abu-Elsaoud: Suez Canal University, Faculty of Science, Botany & Microbiology Department, 41522 Ismailia, Egypt; abuelsaoud@science.suez.edu.eg; orcid.org/0000-0002-6269-3418

Sahar Ahmed El-Shatoury: Suez Canal University, Faculty of Science, Botany & Microbiology Department, 41522 Ismailia, Egypt; sahar_hassan@science.suez.edu.eg; orcid.org/0000-0002-6093-5145

Corresponding author

[Mohamed Diab: (+201000087158) (mohamed.diab_pgs@science.suez.edu.eg)]

Supplementary information

Supplementary S2. Normality testing of study variables using Shapiro wilk and Kolmogorov Smirnov.

Normality testing

Normality testing was performed, using Shapiro-Wilk and Kolmogorov-Smirnov at 0.05 level, for detecting parametric and nonparametric variables. The parametric variables included: total protein, α– and β–esterase, and catalase. Phenol oxidases data was nonparametric, and it was represented in a parametric form for better presentation of mean and standard deviation. The analysis was carried out using computer software Statistical Package for Social Science SPSS (IBM-SPSS ver. 29.0 for Mac OS) **(Knapp, 2017)**.

Supplementary Table 2. Normality testing of study variables using Shapiro wilk and Kolmogorov Smirnov.

Tests of Normality					
**variable**	**Kolmogorov-Smirnova**		**Shapiro-Wilk**		**Normality**
	**Statistic**	**Sig.**	**Statistic**	**p-value**	
**Total protein**	0.191	.200*	0.881	0.09	parametric
**Alpha esterase**	0.227	0.087	0.861	0.051	parametric
**Beta esterase**	0.207	0.165	0.927	0.35	parametric
**Phenol oxidases**	0.414	<.001	0.618	<.001	nonparametric
**Catalase**	0.12	.200*	0.971	0.925	parametric

References

Knapp, H. *Introductory Statistics Using SPSS. Second edition. Los Angeles: SAGE* (2017).
